# The Mental Vulnerability of Affordable Housing Residents Based on Structural Equation Model: A Case Study of Fanghe Garden in Guangzhou

**DOI:** 10.3389/fpsyg.2022.851974

**Published:** 2022-05-16

**Authors:** Yuanyuan Zhang, Liling Li, Bo Tang, Lin Lin

**Affiliations:** ^1^School of Resources and Planning, Guangzhou Xinhua University, Guangzhou, China; ^2^School of Architecture and Urban Planning, Guangdong University of Technology, Guangzhou, China; ^3^School of Geography and Planning, Sun Yat-sen University, Guangzhou, China

**Keywords:** affordable housing, mental vulnerability, structural equation model, housing residents, Fanghe Garden

## Abstract

By constructing a “measure-representation-mechanism” research paradigm of mental vulnerability, this manuscript explores the mental vulnerability measures, spatial representations, and influencing factors of affordable housing residents in Fanghe Garden in Guangzhou by combining the mental vulnerability questionnaire (MVQ) and the structural equation model (SEM). First, the residents of Fanghe Garden had a higher mental vulnerability, difficulties in interpersonal interaction, and lower well-being. Second, the behavioral spatial representations of residents’ mental vulnerability were significantly differentiated. Low-rent housing residents were less active and preferred hidden and small places, while economically affordable housing residents were more active, and they concentrated their activity space in the cultural gallery and central square. Residents were highly willing to interact with neighbors within the community, but mainly with similar people. Third, housing condition, community construction, physical condition, neighborhood communication, and housing experience had a significant negative effect on the mental vulnerability of affordable housing residents.

## Introduction

Housing, especially the housing of low-income groups, has become the focus of social attention. As a type of housing provided by the Chinese government for low- and middle-income families, affordable housing is planned and built by the government, and its construction standard, sales price and rent standard are limited to provide living security for the low-income groups. The specific forms include low-rent housing, economically affordable housing, and government-subsidized rental housing. At present, affordable housing has become an important initiative to improve the quality of life of low-income families in China, and affordable housing communities have also become an important part of urban space (Alteneiji et al., 2020).

Western scholars have started their research on affordable housing earlier, mainly from the perspectives of relevant policies and Public-Private Partnership (PPP) models (Alteneiji et al., 2020; Damoah et al., 2020). Some scholars have also used poor areas as examples to explore the financing model of affordable housing and the impact of residential segregation, reflecting the concern of humanism and economic management on the management of affordable housing (Friedman and Rosen, 2020; Jones and Stead, 2020). Research on affordable housing in China began in the 1990s, but early research results were scarce; from 2007 to 2009, affordable housing management was listed as one of the important responsibilities of the government. The number of research results in affordable housing has increased significantly, mostly in terms of policies and systems (Li, 2007; Wang et al., 2016), economic and social impacts (Zheng and Sun, 2011; Zhao, 2014), supply forecasts (Xu, 2018), surrounding facilities (Liu and He, 2016), spatial location (Zheng and Zhang, 2010; Yang et al., 2020), and internal management (Song, 2011; Zhang and Chai, 2019).

In the 21st century, mental problems are among the most serious health problems facing people. With the increasing understanding of “people-centeredness” and “home” in geography (Feng et al., 2015), the mental well-being of affordable housing residents is receiving more and more attention. Mental vulnerability is an expression/characterization of a certain group of people with low frustration resistance and difficulty socializing with others, represented by the mental vulnerability of affordable housing residents. Compared with other people, low-income groups can less resist and adapt to the internal and external living environment. Hence, the study of the mental vulnerability of affordable housing residents is of great significance to determine whether affordable housing residents can successfully integrate into society, overcome difficulties, and obtain living space (Li and Zhou, 2012). In general, few studies have been conducted on the mental vulnerability of affordable housing residents. Most of the studies only analyzed the impact of affordable housing on residents’ psychology based on the layout and location of affordable housing, the construction of external public space, and social relationships in affordable housing communities (Liu et al., 2019). Scholars found that the public services of public rental housing have a positive impact on increasing the willingness of the mobile population to stay, promoting the living consumption and social fusion of low-income residents. In contrast, the better public activity space in the community enables affordable housing residents to be physically healthy and optimistic (Li et al., 2014). In terms of the management mode, the centralized arrangement model of affordable housing may induce social segregation and exclusion, leading to the labeling of social stratification (Zhao, 2014). However, the mixed housing model can meet the social and mental needs of both wealthy and poor residents and create a platform for inter-class interaction (Song, 2011; Li and Li, 2015; Herbst and Lucio, 2016). From the perspective of affordable housing community relations, affordable housing communities can expose disadvantaged groups to negative neighborhood relations and solidify social classes. At the same time, the development of the Internet and big data has reduced the information gap between different groups and brought about a dilution of neighborhood relations (Logan and Glenna, 1994), which has a greater impact on disadvantaged groups such as the unemployed, the disabled, and the physically ill in the community, who have only limited social networks (Guest and Wierzbicki, 1999). From the perspective of community interaction, neighborhood communication in affordable housing shows a large scale and high frequency but a low level of emotional identity (Li and Zhou, 2012). Therefore, effective community management can provide residents with more social opportunities and promote their identity and fusion into the community (Zhang et al., 2018). Neighborhood interactions in affordable housing are mainly influenced by “housing type” and “community factors,” and have little to do with “residents’ individual household characteristics” and “socioeconomic conditions” (Song, 2011).

In summary, most of the extant studies on affordable housing have focused on infrastructure, neighborhood interaction, and management patterns, but less on the mental vulnerability of affordable housing residents. Most of the existing studies on mental vulnerability are purely mental health measures (Logan and Glenna, 1994), and the spatial representation and formation factors of mental vulnerability are less represented. The quantitative and qualitative analyses are not sufficiently combined in terms of research methods, and multidisciplinary cross-sectional research is hardly pronounced. Guangzhou is one of the first cities in China to build affordable housing, and it has a large immigrant population, an abundance of low-income groups, and obvious characteristics of urban poverty. Based on this, this study takes Fanghe Garden in Guangzhou as an example and combines mental vulnerability questionnaire, Structural Equation Modeling (SEM) and Geographic Information System (GIS) to analyze the measurement, spatial representation, and influencing factors of mental vulnerability of affordable housing residents, in order to provide relevant suggestions for the planning and later management of affordable housing communities.

## Case Selection and Data Source

### Case Selection

**FIGURE 1:**
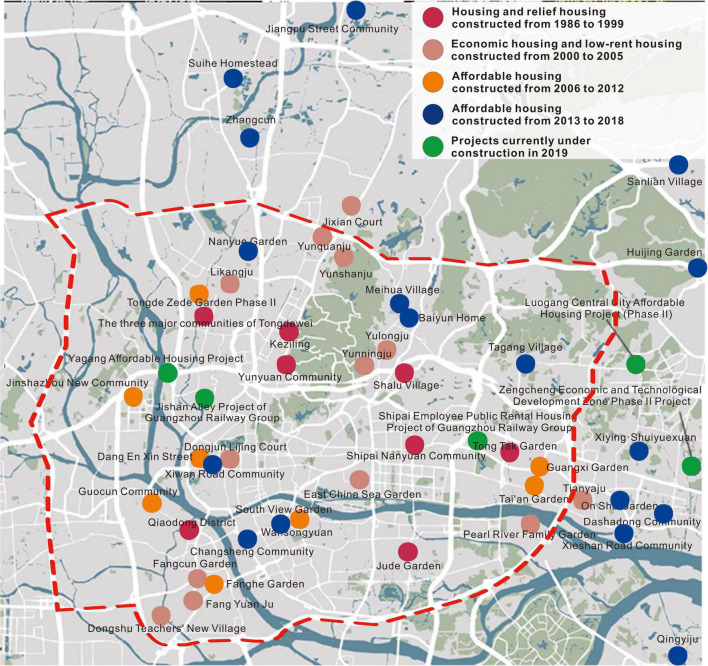
Distribution of affordable housing in the downtown area of Guangzhou.

**FIGURE 2:**
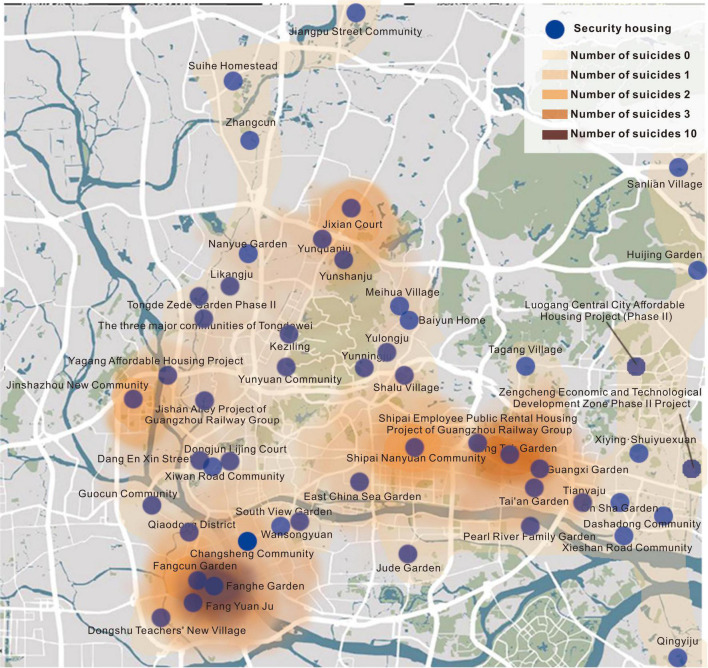
Distribution of suicide rate in affordable housing units in the downtown area of Guangzhou.

There are many affordable housing communities in Guangzhou, and most of the inhabitants are people with difficulties in employment and living. As shown in Figure 1, the current affordable housing communities are mainly located in Tianhe, Haizhu, Liwan, and Yuexiu districts, presenting local clustering characteristics in space. Most of the affordable housing units have experienced social risk events (see Figure 2). Since suicide is an extreme manifestation of mental vulnerability, this manuscript takes the number of suicide cases in affordable housing units as the main indicator for selecting the case site, and Fanghe Garden was selected as the study object. On the one hand, the number of suicide cases in Fanghe Garden is higher than that of other communities, which has attracted widespread social attention. At the same time, Fanghe Garden is a typical high-end mixed community of economically affordable and low-rent housing, with a complex and special structure of internal residents.

### Data Source

The methodology of this study is mainly based on questionnaire survey, supplemented by interview and observation. The questionnaires and interviews were targeted at all residents of Fanghe Garden (a total of 5,935 families with about 23,000 residents, including about 2,568 families in the low-rent housing, accounting for about 43%). However, in order to clarify the questionnaire and interview subjects, the research group used random sampling and follow-up survey. A simple random sampling strategy was used and 100 respondents were selected as the research sample for this manuscript. A total of 100 questionnaires were distributed, and the returned questionnaires included 36 for the low-rent housing and 63 for the affordable housing. Each questionnaire included 2 tables, Tables 1, 2, which were distributed simultaneously. Table 1 is a measure of mental vulnerability of the residents, which mainly measures the basic status of mental vulnerability of the residents of Fanghe Garden. Table 2 is a scale of factors influencing mental vulnerability, which mainly summarizes the disturbing factors of mental vulnerability of the residents of Fanghe Garden. The validity rate of the questionnaire reached 95%, which meets the requirements of the questionnaire. The sample size is between 30 and 460, which meets the required range of SEM sample size (Wolf et al., 2013). A semi-structured interview was also conducted with 10 people, focusing on the residents’ experience of living in the community. The survey period was from April to July 2019.

**TABLE 1:** Measure scale of community residents’ mental vulnerability.

Dimension		Questions
Physical and mental symptoms	P1	Your hands easily tremble
	P2	You often lose your appetite
	P3	You have frequent headaches
	P4	You have frequent insomnia
	P5	You have frequent anxiety attacks
	P6	You often feel very tired
Psychiatric symptoms	C1	You often take medication, such as headache pills, sleeping pills, sedatives
	C2	You often suffer from severe nervousness
	C3	You often feel dizzy
	C4	You are in a bad mood almost all the time
	C5	If someone is watching you, you can hardly concentrate on your work
	C6	You have trouble making friends
Interpersonal problems	A1	You find it hard to accept other people’s decisions about you
	A2	You prefer to be yourself
	A3	You get angry and upset over minor things
	A4	You often have thoughts that bother or worry you
	A5	You are extremely shy or sensitive
	A6	You often feel misunderstood by others

**TABLE 2:** List of factors influencing the mental vulnerability of community residents.

Category	Sub-item	Indicator name	Description
Living conditions	Housing condition	Density	1 = very unimportant, 5 = very important, 5-point Likert scale
		Height	
	Housing experience	Ventilation	1 = very unimportant, 5 = very important, 5-point Likert scale
		Lighting	
		Space sense in the hallways	
		Green building materials	
		Building quality	
Community environment	Community building	Internal public space	1 = very unimportant, 5 = very important, 5-point Likert scale
		Public services	
		Law and order	
		Medical and health facilities	
		Overall color	
		Internal roads	
		Cultural activities	
	Internal interaction	Interaction with residents of the same unit building	1 = very unimportant, 5 = very important, 5-point Likert scale
	Neighborhood interaction	Interaction with residents of the same community	1 = very unimportant, 5 = very important, 5-point Likert scale
Surroundings	Fusion with surrounding community residents	Deeper engagement with residents in the surrounding community	1 = very unimportant, 5 = very important, 5-point Likert scale
		Friendship with residents in the surrounding community	
		Common neighbors with residents in the surrounding community	
	Location of community spaces	Number of transportation facilities within a 20-min walk	1 = very unimportant, 5 = very important, 5-point Likert scale
		Number of public services within a 20-min walk	
Personal conditions	Physical conditions	Health status	1 = very unimportant, 5 = very important, 5-point Likert scale
		Fitness status	

*Remarks: 1. Special families refer to special families such as widowed elderly, disabled, martyrs’ families, etc.; 2. Immediate families refer to three generations and above residing together in the same housing; 3. The standard of families with minimum guarantee: The per capita monthly income of families is lower than the minimum living guarantee standard of the city (Guangzhou’s minimum living guarantee standard is RMB 1010/month).*

The characteristics of the sample of Fanghe Garden in this survey: (1) The residents are mainly middle-aged and elderly, and the proportions of men and women are equal; (2) In terms of family structure, 19% are single-person families and 30% are immediate families; (3) Regarding marital status, 36% of the residents are single, divorced, or widowed; (4) Residents’ education level is generally low, with only 21% of them having a bachelor’s degree or above; (5) In terms of employment, 52% of the residents have no fixed jobs or are retired; (6) Low- and middle-income families with a monthly household income of less than 3,500 yuan/month account for about 50% of the residents, and their income is low; (7) Among the household categories, low-income families, special families, people eligible for subsistence allowances, and people in extreme poverty account for about 50% of the total. These sample characteristics cover a wide range and are typical for this study on the mental vulnerability of the residents in affordable housing.

## Research Methodology

### Mental Vulnerability Scale

The mental vulnerability Questionnaire developed by Eplov has been proved to offer a more accurate prediction of the physical and mental status of the population (Eplov et al., 2010), and it has been used in several empirical studies with high reliability and validity (Sequeira et al., 2017). This manuscript uses a modified version of the questionnaire based on the actual situation in China (Guo et al., 2019). A pre-survey was conducted on the case location using this questionnaire and then the questionnaire was modified depending on the actual situation of the affordable housing community surveyed. The final formal questionnaire was developed with 18 items for physical and mental symptoms (6 items), psychiatric symptoms (6 items), and interpersonal issues (6 items) to get an idea of the mental status of the residents in the affordable housing community. According to the Likert 5-point scale, respondents were asked to evaluate it according to their actual situation.

### Structural Model

Structural equation modeling is a validated multivariate statistical analysis technique that has become an important analytical tool within quantitative research. Multiple indicator areas are used to reflect latent variables and to analyze the relationships between concepts (factors) throughout the model (Hou and Cheng, 1999; Cheng, 2006). The current study used AMOS software to develop a structural equation model and explored the main causes affecting the mental vulnerability of the residents of Fanghe Garden in combination with SPSS 20.0.

#### Establishing Impact Factor Indicators

Based on the related research results (Chen et al., 2015), this manuscript mainly investigates the influence of housing condition, housing experience, community construction, neighborhood interaction, fusion with neighboring residents, and personal status of the affordable housing residents on the mental vulnerability of community residents, and creates a table of factors influencing the mental vulnerability of community residents. We included indicators such as housing experience and community environment in the index system. The questionnaire is to determine the status and importance of the influence of the current housing environment and community environment on the mental vulnerability of the residents. What is investigated are the actual feelings and perceptions of the respondents, not the mental expectations of housing, so the indexes can be considered as factors affecting the mental vulnerability of the residents. In addition, unit type, housing area, housing type, personal attributes, and family characteristics, as the basic information of respondents, are not included in the establishment of the influence mechanism.

#### Model Determination

Based on previous studies and our team’s field research, the positive and negative effects of exogenous variables on endogenous variables were assumed, and a structural equation model was constructed in this manuscript as shown below, with the following hypotheses:

H1: Housing condition has a significant negative effect on mental vulnerability.

H2: Housing experience has a significant negative effect on mental vulnerability.

H3: Community building has a significant negative effect on mental vulnerability.

H4: Neighbor interaction within the community has a significant negative effect on mental vulnerability.

H5: Fusion with residents in the surrounding communities has a significant negative effect on mental vulnerability.

H6: Spatial location has a significant negative effect on mental vulnerability.

H7: Physical status has a significant negative effect on mental vulnerability.

## Structural Model Analysis

### Confirmatory Factor Analysis of the Mental Vulnerability Measure for Residents of Fanghe Garden

The indicators were rated from 1 to 5 in ascending order. The mean value of mental vulnerability of the residents of the affordable housing in Fanghe Garden was 3.35, with a moderately high level of vulnerability (greater than the mean of the maximum and minimum values of 3.11) and a standard deviation of 0.88. From the specific measures of “physical and mental,” “mental,” and “interpersonal” (Table 3), it can be seen that community residents scored higher in the “interpersonal” dimension, which means that this community group has certain difficulties in interacting with others. In the “mental” dimension, the average value of the community was 3.49, with a general situation and a low level of mental adaptation. In particular, the mean values of “nervousness,” “dizziness,” and “bad mood” were higher than the mean scores of this dimension, indicating that the well-being of residents in Fanghe Garden was low. In the “physical and mental” dimension, the mean value of this community group was 3.22, which showed that they felt “a loss of appetite,” “headache,” “insomnia,” and “very tired.”

**TABLE 3:** Residents’ mental vulnerability in the affordable housing community of Fanghe Garden.

	Comprehensive measure	Physical and mental	Mental	Interpersonal
Min.	1.33	1.00	1.00	1.00
Max.	4.89	5.00	5.00	4.83
Mean	3.35	3.22	3.49	3.35
Standard deviation	0.88	1.05	1.04	1.08
Skewness	−0.69	−0.09	−0.63	−0.50
Kurtosis	−0.60	−0.96	−0.70	−1.07

In general, the residents of Fanghe Garden had more difficulties in interpersonal interactions and showed chronic mental tension and physical and mental discomfort, which to a certain extent indicates the more pronounced mental vulnerability of the residents of Fanghe Garden.

Confirmatory factor analysis was further conducted on the corresponding scale of mental vulnerability of community residents in Fanghe Garden, as shown in Table 4. The KMO test value of the model was 0.949 (>0.7) and the Bartlett’s spherical test value was 3881.350 (*p* < 0.001), which indicated that the mental vulnerability scale was suitable for factor analysis. The results of the confirmatory factor analysis are presented in the table below.

**TABLE 4:** Confirmatory factor analysis table.

			Estimate	S.E.	C.R.	*P*
Physical and mental	→	Physical and mental 1	1			
Physical and mental	→	Physical and mental 2	1.733	0.434	3.992	***
Physical and mental	→	Physical and mental 3	2.016	0.464	4.346	***
Physical and mental	→	Physical and mental 4	1.236	0.348	3.55	***
Physical and mental	→	Physical and mental 5	1.108	0.304	3.646	***
Physical and mental	→	Physical and mental 6	1.572	0.401	3.915	***
Psychological	→	Psychological 1	1			
Psychological	→	Psychological 2	0.788	0.185	4.26	***
Psychological	→	Psychological 3	1.537	0.286	5.369	***
Psychological	→	Psychological 4	0.581	0.206	2.824	0.005
Psychological	→	Psychological 5	1.828	0.476	3.841	***
Psychological	→	Psychological 6	0.268	0.22	3.216	***
Interpersonal	→	Interpersonal 1	1			
Interpersonal	→	Interpersonal 2	0.147	0.216	3.679	***
Interpersonal	→	Interpersonal 3	1.344	0.339	3.968	***
Interpersonal	→	Interpersonal 4	0.398	0.221	2.802	***
Interpersonal	→	Interpersonal 5	0.542	0.242	2.239	0.025
Interpersonal	→	Interpersonal 6	0.888	0.254	3.492	***

***Indicates that the specific value will be displayed when p < 0.001, P > 0.001, and the path is significant when p < 0.05.

From the confirmatory factor analysis table, i.e., the scale information table, we can see that the Cronbach’s α of each factor was greater than 0.7, and according to the judgment interval of the Cronbach’s α value, we can judge that the scale had high reliability. The table also shows the standardized factor loadings of the measures in the confirmatory factor analysis on the variables of interest as well as the AVE (average variance extraction) and CR (combined reliability) values for each construct. The CR values were greater than 0.7, indicating that the scale had good composite reliability. Meanwhile, the AVE of each variable was greater than 0.5, which implied that the scale had good convergent validity.

### Behavioral Spatial Representation of Mental Vulnerability of Affordable Housing Residents

The project team members conducted observation and research at different times, and interviewed residents about their willingness to choose activity space and activity frequency. Based on the observation data and interview results, this study analyzed the characteristics and causes of activity behavior of different groups of people in affordable housing communities, and the results are as follows.

#### Low-Rent Housing Residents Are Less Active and Prefer Isolated and Small Spaces

The results of the follow-up study show that the spatial distribution of residents’ activities in Fanghe Garden shows the spatial segregation of different types of families compared with the general commodity housing complex. There are two categories of affordable housing residents and low-rent housing residents in Fanghe Garden. Affordable housing residents are in a well-off financial situation, while low-rent housing residents are among the low-income groups living in difficult conditions.

**FIGURE 3:**
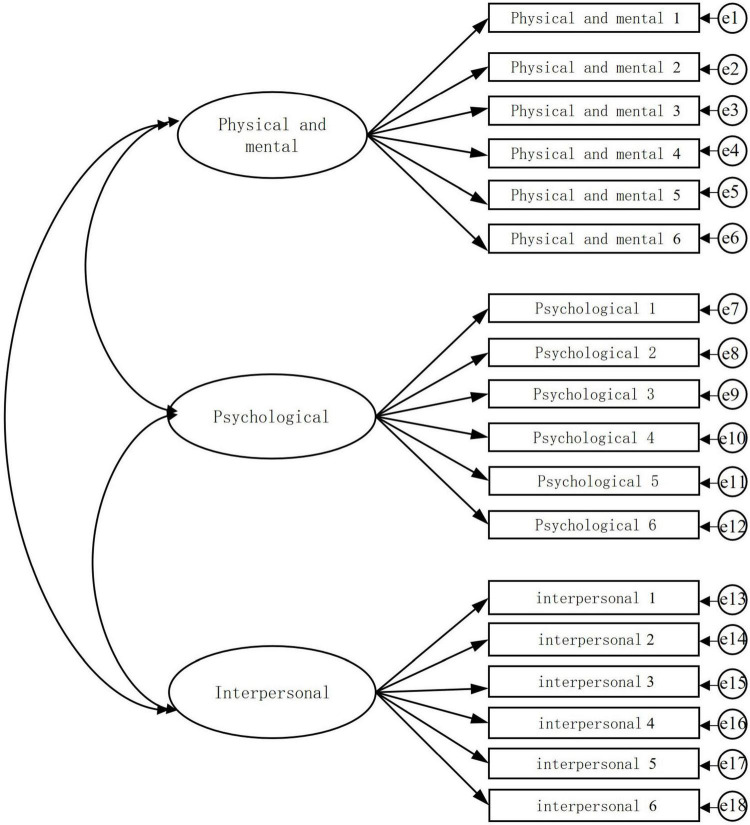
Confirmatory factor analysis of mental vulnerability.

**FIGURE 4:**
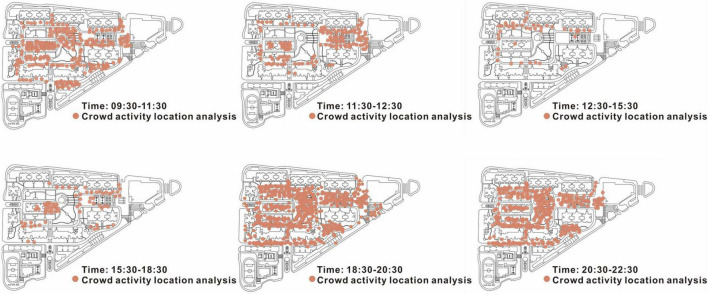
Analysis of the locations of crowd activities in different periods.

Based on the field observation, the members of the research group made a map of the location distribution of people’s activities in different time frames based on the interview and observation data, as shown in Figure 3. The residents of affordable housing in Fanghe Garden are more active in the morning from 9:30 to 11:30 and in the evening from 18:30 to 22:30, with a higher frequency of going out. Their activity spaces are concentrated in the cultural corridor and the central square, while others are scattered in the fitness area of the community and the children’s facilities area. Through the observation records, we found that the users of the public space in the community are mainly elderly people and children, followed by young people from other commodity housing complexes in the surroundings. In Figure 4, one of the main reasons for the residents who are more active between 18:30 and 20:30 is that many young men from the commodity housing in the surroundings enter the community to play on its courts. However, in further interviews, we found that these “outsiders” do not exercise or communicate with the residents of affordable housing in this community. In the interviews with two of the outsiders, they said that the residents in Fanghe Garden are more wary of and reject outsiders, so they do not initiate communication with the residents there. During the interviews with the community administrators, we noticed that there is in fact no lack of young people in Fanghe Garden, and that these people are often qualified to move in because of their difficult family backgrounds and low income from their jobs. However, we seldom saw them staying or talking with others during our observation of the public spaces in the community.

In our study of low-rent housing residents and affordable housing residents in this community, we found that they presented different characteristics in terms of activity space location, activity content, and communication with others. Of the 36 completed questionnaires for low-rent housing residents, 89% of them chose to spend more of their time in the residential open space and nearby paths at the edge of the community, tending to choose spaces that were more remote and secluded. From our observations, we noted that most of their activities were to chat with the residents in the same low-rent housing area in a low voice, and that they were less likely to show up in the more crowded public places in the middle of the community. In contrast, the majority of affordable housing residents chose the open public areas in the middle of the community, such as the cultural corridor and the central square. Among them, the elderly are the main users, preferring sports and fitness activities such as tai chi, table tennis and dancing, as well as social activities such as gossiping and playing cards. The frequency of outings for recreational activities was also lower among low-rent housing residents as compared to affordable housing residents. We organized the interviews and discovered that on the one hand, this is because low-rent housing communities are somewhat farther away from places of activity than affordable housing. On the other hand, in the interviews, low-rent housing residents expressed an increased need for seclusion and wariness of the outside world, and therefore they were less likely to be seen in public areas. Based on interviews with some low-rent housing residents, it was found that low-rent housing residents are more inclined to convey signals of their families’ difficulties to the outside world because of the higher eligibility requirements for low-rent housing and the social scrutiny of its fairness. Some of the older residents of low-rent housing even indicated that when they were in public spaces, they avoided such behaviors that might seem pleasant to others, like “playing cards” and “laughing loudly” as much as possible. From the state of conversation with the researcher, the residents of the affordable housing showed a more open attitude, while we could feel their clear caution and rejection in our communication with the residents of the low-rent housing. They also expressed more complaints, sadness, and other emotions during the interviews, and were more likely to talk about all kinds of difficulties of their families.

According to policies on affordable housing and low-rent housing in Guangzhou, the eligibility requirements for affordable housing require the per capita disposable income of the household to be less than 80% of the per capita disposable income of urban residents in the previous year, while the eligibility requirements for low-rent housing require the per capita monthly income of the household to be less than 800 yuan, which can be considered an impoverished household under the current price level. From the housing rent of affordable housing and low-rent housing, according to the public rental housing rent in Guangzhou in 2019, the monthly rent of the elevator building of the affordable housing in Fanghe Garden is 30 yuan/square floor area, while the monthly rent of low-rent housing starts from a standard of only 1 yuan/square floor area. Therefore, the review of the eligibility for low-rent housing will be extremely stringent, and the government has also developed very stringent supervision methods for admission and withdrawal. The review and supervision of the eligibility for low-rent housing is of great concern to society. In general, there are more negative labels attached to low-rent housing tenants in social perceptions, such as “poor,” “sad,” “inferior,” and other negative impressions. In both interviews and observations, we saw that low-rent housing tenants were deliberately catering to these stereotypes in order to retain their qualifications for low-rent housing. At the same time, such environments and self-suggestions further reinforce the mental vulnerability of low-income groups.

#### High Willingness to Interact With Neighbors Within the Community, but Mainly With Similar Individuals

As shown in Table 5, the residents of Fanghe Garden engage in more activities in the public space, so there are more opportunities for neighborhood interaction. Spatially, the cultural gallery on the elevated floor of the ground floor of buildings 19, 20, and 21 attracts more activity. In addition, the residents also concentrate their activities in the fitness facilities inside the central square, the children’s activity area, other leisure and recreational places, and the neighborhood roads. Among them, the fitness facilities are mainly used by teenagers. Children mainly use the children’s activity area. The roads in the neighborhood mainly function as dog walking and running.

**TABLE 5:** Analysis of crowd activities in the residential area of Fanghe Garden.

Public activity space		User	Activity content
Dotted space	Enclosed space	Surrounding residents	Housekeeping activities, small talk, etc.
	Building entrance	Families, close neighbors	Spending money, eating and drinking tea, chatting, etc.
	Public service space	Vendors, community members, passers-by	Spending money, talking
Linear space	Street space	Community residents, passers-by	Walking, dog walking, strolling, trading, gossip, childcare, observation, etc.
	Alley space	Community residents	Traffic function mainly, little conversation, walking, etc.
Surface space	Attached to a school	Students mainly, some community residents	Exercise, walking, chatting, etc.
	Attached to residence	Residents in the vicinity of the space	Gossiping, talking, playing chess, dancing, playing shuttlecock, playing table tennis, playing tai chi, chorus, walking the dog, taking care of children, etc.
	Attached to the street	Residents in the vicinity of the space, passers-by, etc.	Gossiping, playing chess, walking the dog, taking care of children, children playing, etc.

The questionnaire statistics and further interviews revealed that less than 35% of the residents considered it more important to be friends with the residents of the neighboring commodity housing. However, when asked about their interactions with residents in the same unit or neighborhood, over 80% of residents agreed that interactions with neighbors were essential, and the conventional belief that “an afar off relative is not as helpful as a near neighbor” was generally accepted. In-depth interviews on the choice of people to interact with on a daily basis showed that most residents generally interacted more frequently with neighbors who were closer to their family environment, had more common topics to discuss, and had more empathy on the same issues. In the interviews, when asked whether economically affordable and low-rent housing residents would become friends with low-rent housing residents, most economically affordable housing residents said it was difficult. In contrast, low-rent housing residents were more likely to associate with people who were also low-rent housing residents. Economically affordable housing residents have a preconceived notion that low-rent housing residents are negative and difficult to associate. For low-rent housing residents, their friends who are also in financial difficulties will be more empathetic to their situation and more likely to have emotional support for each other.

Generally speaking, the behavior of Fanghe Garden residents is mainly confined to the public facilities and housing within the community, and the similar group of people also dominates their communication within the family and the community. Then, the analysis of their mental vulnerability can focus on the two factors of “home” and “community building.”

### Analysis of the Influence Mechanism of Mental Vulnerability of Residents of Fanghe Garden

#### Confirmatory Factor Analysis of Influencing Factors

**FIGURE 5:**
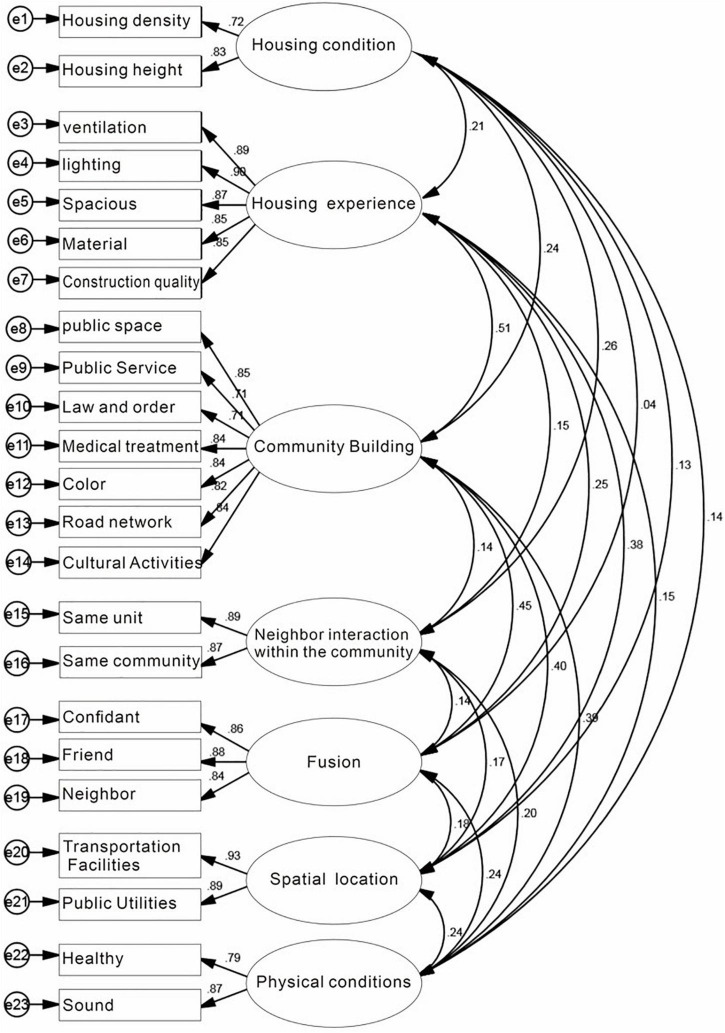
Diagram of the confirmatory factor model.

The confirmatory factor model is shown in Figure 5, the goodness-of-fit of the structural equation model is reflected by the relative chi-square (CMIN/DF), goodness-of-fit index (GFI), root mean square of error of approximation (RMSEA), root mean square of residuals (RMR), adjusted goodness-of-fit index (AGFI), normed fit index (NFI), and Tucker-Lewis Index (TLI). In this manuscript, the goodness-of-fit of the structural equation model was verified in terms of both independent and dependent variables, respectively. For the independent variables, as shown in Figure 3 and Table 6, CMIN/DF was 1.266, which is less than 3 (standard), GFI = 0.927, AGFI = 0.903, which is greater than 0.8 (standard), TLI, IFI, and CFI all reached 0.9 (standard), and RMSEA was 0.030, which is less than 0.08 (standard). It indicates that most of the fit indexes meet the criteria of general SEM studies.

**TABLE 6:** Scale information table.

			Standardized factor loading	Cronbach α	CR	AVE
Physical and mental	→	Physical and mental 1	0.752	0.731	0.732	0.638
Physical and mental	→	Physical and mental 2	0.867			
Physical and mental	→	Physical and mental 3	0.881			
Physical and mental	→	Physical and mental 4	0.824			
Physical and mental	→	Physical and mental 5	0.751			
Physical and mental	→	Physical and mental 6	0.737			
Psychological	→	Psychological 1	0.711	0.751	0.75	0.614
Psychological	→	Psychological 2	0.768			
Psychological	→	Psychological 3	0.745			
Psychological	→	Psychological 4	0.736			
Psychological	→	Psychological 5	0.779			
Psychological	→	Psychological 6	0.839			
Interpersonal	→	Interpersonal 1	0.716	0.769	0.771	0.539
Interpersonal	→	Interpersonal 2	0.781			
Interpersonal	→	Interpersonal 3	0.712			
Interpersonal	→	Interpersonal 4	0.725			
Interpersonal	→	Interpersonal 5	0.788			
Interpersonal	→	Interpersonal 6	0.732			

#### Influence Mechanism Analysis of Mental Vulnerability

**FIGURE 6:**
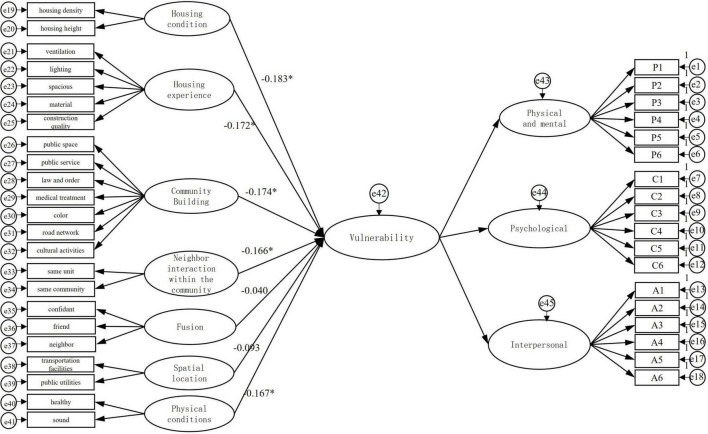
Structural results of mental vulnerability model.

As shown in Figure 6 and Table 7, the housing condition of residents has a significant negative effect on their mental vulnerability (*p* < 0.05), and for every 1 unit increase in housing condition, the mental vulnerability of residents will decrease by 0.183 units. Therefore, the H1 hypothesis is valid. The density and height of the housing directly affect the privacy of the residents and the physiological condition of the residents (e.g., acrophobia, etc.), resulting in physical and mental discomfort and a sense of depression among the residents. In the long run, it has a certain effect on the mental vulnerability of the residents.

**TABLE 7:** Path analysis results.

Path relationship			Standardized coefficient	Unstandardized coefficient	Standard error	*T*-value	*P*	Support for the hypothesis (Yes/No)
Vulnerability	←	Housing condition	–0.183	–0.115	0.049	–2.343	0.019	Yes
Vulnerability	←	Housing experience	–0.172	–0.147	0.062	–2.384	0.017	Yes
Vulnerability	←	Community building	–0.174	–0.154	0.075	–2.052	0.04	Yes
Vulnerability	←	Neighbor interaction within the community	–0.166	–0.121	0.047	–2.554	0.011	Yes
Vulnerability	←	Fusion	–0.04	–0.034	0.058	–0.597	0.551	No
Vulnerability	←	Spatial location	–0.093	–0.061	0.045	–1.359	0.174	No
Vulnerability	←	Physical conditions	–0.167	–0.163	0.071	–2.305	0.021	Yes

The hypothesis of H3 is valid because the mental vulnerability of residents decreases by 0.174 units for every 1 unit increase in community building (*p* < 0.05). Based on the public services of the residents, community building reduces the mental vulnerability by improving the public services and building public open space, so that the residents can have an effective platform to communicate with each other and improve the satisfaction of living in the community (Figure 6 and Table 7). The survey found that most of the residents of Fanghe Garden are active inside the community, and the community building is the hardware facilities for the residents to carry out activities, which should be reasonably arranged and meet the residents’ basic needs.

Housing experience has a significant negative effect (*p* < 0.05) on the mental vulnerability of affordable housing residents, with a standardized factor loading coefficient of −0.172, and thus the H2 hypothesis is valid. The factor loadings of “lighting” and “ventilation” were 0.90 and 0.89, respectively, more influential. Residents are exposed to light and ventilation every day, and their construction has a great influence on the perception of community residents. If the residents are in a dark environment for a long time, it may lead to health problems and depression, affecting their mental vulnerability. By improving the ventilation and lighting in the community, the mental vulnerability of affordable housing residents can be alleviated and the negative depression can be reduced.

In terms of physical conditions, there is a significant negative effect (*p* < 0.05) on the mental vulnerability of affordable housing residents with a standardized factor loading coefficient of −0.167, so hypothesis H7 is valid. Since affordable housing is for low and middle-income people, their economic conditions are generally poor, which causes some pressure on the residents themselves. Physical conditions have a direct impact on the positive and negative emotions of the population. By improving the physical health and well-being of the population, the burden and risk of the family can be effectively reduced, and therefore the impact of stress in real life on the mental vulnerability of the population can be relatively reduced.

In terms of neighbor interaction within the community, there is a significant negative effect on the mental vulnerability of affordable housing residents (*p* < 0.05), with a standardized factor loading coefficient of −0.166, so the H4 hypothesis is valid. Homogeneous neighborhood communication allows affordable housing residents to share the same feelings and reduce the perception of being “different.”

In addition, fusion with residents in the surrounding community and the community’s location, although significantly and negatively correlated with vulnerability, did not play a significant role. This is because affordable housing residents perceive that interaction with neighbors in the same community is more important for achieving a sense of “commonality” than interaction with residents in the surrounding community. In addition, residents have adequate knowledge of the location of the affordable housing community before they move in, as they need to apply and be approved to be eligible. Therefore, after moving in, residents do not experience significant mental fluctuations due to the location, and there is less impact on their mental vulnerability.

## Conclusion, Recommendations, and Discussion

### Conclusion

(1) According to the measurement results of mental vulnerability of affordable housing residents, the affordable housing residents of Fanghe Garden have more pronounced mental vulnerability. In terms of physical and mental symptoms, most of the residents of Fanghe Garden have symptoms such as “loss of appetite,” “headache,” and “insomnia.” In terms of psychiatric symptoms, most of the residents experience “nervousness” and “dizziness,” and most of them have a low sense of well-being. In terms of interpersonal problems, the residents in this community have little communication with their neighbors and the surrounding community.
(2) Fanghe Garden is a typical mixed community of economically affordable housing and low-rent housing, where the behavioral representations of residents’ mental vulnerability are more clearly differentiated. Residents of low-rent housing are less active and prefer hidden and small places, while residents of economically affordable housing are more active and their activity spaces are concentrated in the cultural gallery and the central square. Although residents have a high willingness to interact with neighbors and have a rich variety of activities, they interact mainly with similar groups. Therefore, by analyzing the causes of mental vulnerability, we can focus on the two factors of “home” and “community building.”
(3) SEM was used to explore the mechanisms and effects of mental vulnerability of affordable housing residents, and it was found that a combination of factors caused the mental vulnerability of affordable housing residents. The five factors of housing condition, community building, physical conditions, neighborhood communication with residents in the community, and housing experience were found to significantly negatively affect the mental vulnerability of affordable housing residents in Fanghe Garden.

### Recommendations

(1) Public construction within affordable housing communities should be strengthened. The interaction between neighbors acts on certain community building and activity sites. Then, in the future, affordable housing should give more consideration to creating a positive community atmosphere and improving public service facilities to truly meet the needs of affordable housing residents for neighborhood communication. Given that a large proportion of affordable housing residents are elderly, we need to pay more attention to recreation, sports, and space for residence in the process of construction and improvement, and we need to consider the composition of the community residents in the shaping of recreation, sports, and leisure spaces, and build spaces for activities suitable for the elderly.
(2) Community activities should be increased to promote resident engagement. As communities become more important in grassroots services, they have become an important hub for social fusion. Therefore, it is necessary to give full play to the power of organizations in the construction of affordable housing, enhance community cohesion through cultural exhibitions, knowledge education and other activities, promote the participation of residents, and enhance the residents’ sense of belonging to “home.”
(3) Building height and density should be reasonably set to improve the comfort of residents’ housing. The height and density of housing influence the psychology of individuals to a certain extent. Therefore, in the community building process of affordable housing, it is necessary to set a reasonable building height and density to create a pleasant living environment and make the “home” more comfortable.

### Discussion

The well-being of affordable housing residents serves as an important driving force for community building and management. This manuscript explored the mental vulnerability of residents in Fanghe Garden from the perspective of mental vulnerability of affordable housing residents by constructing a research paradigm of “measure-representation-mechanism” of mental vulnerability, combining mental vulnerability questionnaire, and SEM. Thus, it provides theoretical significance and case support for the development and planning management of low-rent housing communities. However, since the measurement and influence mechanism of mental vulnerability involves different dimensional factors, the analysis using sample questionnaires and SEM alone is not comprehensive enough, and the number of questionnaires can be increased further to refine the research method in the future study. At the same time, this manuscript summarized the differences in the mental vulnerability representations of low-rent housing and economically affordable housing residents, but the factors influencing the differences in mental vulnerability were not analyzed in depth. These influencing factors contribute to creating an environment and interpersonal harmony within affordable housing. It will be one of the focuses of future research.

## Data Availability Statement

The original contributions presented in the study are included in the article/supplementary material, further inquiries can be directed to the corresponding author.

## Ethics Statement

Ethical review and approval was not required for the study on human participants in accordance with the local legislation and institutional requirements. Written informed consent from the patients/participants was not required to participate in this study in accordance with the national legislation and the institutional requirements.

## Author Contributions

YZ and BT contributed to the development of the framework and writing of the manuscript, figures, and the literature review. LilL contributed to analysis and interpretation of data for the work. LinL contributed to guide the text improvement of the manuscript. All authors have read and approved the final manuscript.

## Conflict of Interest

The authors declare that the research was conducted in the absence of any commercial or financial relationships that could be construed as a potential conflict of interest.

## Publisher’s Note

All claims expressed in this article are solely those of the authors and do not necessarily represent those of their affiliated organizations, or those of the publisher, the editors and the reviewers. Any product that may be evaluated in this article, or claim that may be made by its manufacturer, is not guaranteed or endorsed by the publisher.
